# Post-viral symptoms and conditions are more frequent in COVID-19 than influenza, but not more persistent

**DOI:** 10.1186/s12879-024-10059-y

**Published:** 2024-10-09

**Authors:** Falko Tesch, Franz Ehm, Friedrich Loser, Lars Bechmann, Annika Vivirito, Danny Wende, Manuel Batram, Tilo Buschmann, Simone Menzer, Marion Ludwig, Martin Roessler, Martin Seifert, Giselle Sarganas Margolis, Lukas Reitzle, Christina König, Claudia Schulte, Dagmar Hertle, Pedro Ballesteros, Stefan Baßler, Barbara Bertele, Thomas Bitterer, Cordula Riederer, Franziska Sobik, Christa Scheidt-Nave, Jochen Schmitt

**Affiliations:** 1 Center for Evidence-Based Healthcare (ZEGV), University Hospital and Faculty of Medicine Carl Gustav Carus, TU Dresden, Fetscherstraße 74, Dresden, 01307 Germany; 2 Techniker Krankenkasse, Bramfelder Straße 140, Hamburg, 22305 Germany; 3 IKK classic, Tannenstraße 4b, Dresden, 01099 Germany; 4 InGef - Institute for Applied Health Research Berlin GmbH, Otto-Ostrowski-Straße 5, Berlin, 10249 Germany; 5 BARMER Institut Für Gesundheitssystemforschung (bifg), Axel-Springer-Straße 44, Berlin, Germany; 6 Vandage GmbH, Detmolder Str. 30, Bielefeld, 33604 Germany; 7 AOK PLUS, Sternplatz 7, Dresden, 01067 Germany; 8 Robert Koch Institute, Nordufer 20, Berlin, 13353 Germany; 9 DAK-Gesundheit, Großer Burstah 23, Hamburg, Germany

**Keywords:** SARS-CoV-2, Post-COVID, Influenza, Cohort study, Claims data

## Abstract

**Background:**

Post-viral symptoms have long been known in the medical community but have received more public attention during the COVID-19 pandemic. Many post-viral symptoms were reported as particularly frequent after SARS-CoV-2 infection. However, there is still a lack of evidence regarding the specificity, frequency and persistence of these symptoms in comparison to other viral infectious diseases such as influenza.

**Methods:**

We investigated a large population-based cohort based on German routine healthcare data. We matched 573,791 individuals with a PCR-test confirmed SARS-CoV-2 infection from the year 2020 to contemporary controls without SARS-CoV-2 infection and controls from the last influenza outbreak in 2018 and followed them up to 18 months.

**Results:**

We found that post-viral symptoms as defined for COVID-19 by the WHO as well as tissue damage were more frequent among the COVID-19 cohort than the influenza or contemporary control cohort. The persistence of post-viral symptoms was similar between COVID-19 and influenza.

**Conclusion:**

Post-viral symptoms following SARS-CoV-2 infection constitute a substantial disease burden as they are frequent and often persist for many months. As COVID-19 is becoming endemic, the disease must not be trivialized. Research should focus on the development of effective treatments for post-viral symptoms.

**Supplementary Information:**

The online version contains supplementary material available at 10.1186/s12879-024-10059-y.

## Background

Post-viral symptoms have long been known in the medical community, but more recently received public attention due to the COVID-19 pandemic [1]. Post-COVID condition include a wide variety of post-viral symptoms and conditions resulting from SARS-CoV-2 infection that affect many people worldwide, even after the pandemic has ended. Although several clinical trials are currently underway, there are currently no approved therapies for patients with post-COVID condition [2]. The number of patients with post-COVID condition depends on the proportion of infected individuals developing post-COVID condition and on the persistence of the underlying symptoms.

Evidence suggests that most post-COVID symptoms and conditions resolve within one year from diagnosis [3]. However, various longer-lasting symptoms may severely interfere with daily life activities. One study estimated that over a two-year period, post-COVID condition cumulatively caused about 80.4 and 642.8 disability-adjusted life years (DALYs) per 1,000 persons among non-hospitalized and hospitalized individuals, respectively [4].

Estimates of symptom prevalence and persistence vary substantially due to heterogeneous study designs, follow-up periods, syndrome definitions, and data collection tools. So far, many studies have been restricted to selected groups (e.g. hospitalized patients) or specific outcomes (e.g. fatigue), or are limited due to the lack a comparison group. This makes it difficult to distinguish symptoms related to post-COVID condition from symptoms that would also have occurred in the absence of SARS-CoV-2 infection [5].

In a previous analysis of a matched cohort study based on routine healthcare data we estimated that 15.1% of adults suffer from post-COVID symptoms and conditions three to six months after the acute SARS-CoV-2 infection [6]. A meta-analysis pooling 56 studies estimated the proportion of three post-COVID symptom and condition clusters three months after symptomatic SARS-CoV-2 infection. The three clusters were persistent fatigue with physical pain or mood swings, cognitive problems or ongoing respiratory problems. Overall, 6.2% of patients suffered from at least one of these clusters after three months and 0.9% after 12 months [7].

Post-viral symptoms are not limited to SARS-CoV-2 infection. Other viruses, such as influenza, have the potential to cause post-viral symptoms [1]. In particular chronic fatigue syndrome (CFS) has long been associated with viral infections, such influenza. Influenza has been shown to cause more frequent CFS than other viruses as Varicella-zoster virus or Candida [8]. There is currently ongoing scientific debate regarding the specificity, severity, frequency and persistence of post-viral symptoms related to SARS-CoV-2 compared to other viral infections.

We undertook a large population-based cohort study to investigate whether adults with an infection of the ancestral variant of SARS-CoV-2 compared to adults with influenza suffer more frequently and/or more persistently from post-viral symptoms. In addition to a matched cohort of individuals with influenza from 2018, our study includes a contemporary control cohort of persons not affected by SARS-CoV-2 in the same time span.

## Methods

### Study design

We conducted a matched cohort study based on routine health care data as applied in a previous study [6]. In the present analysis, we compared the rates of newly diagnosed symptoms and conditions between adult individuals with and without documented SARS-CoV-2 infection and influenza infection based on ICD-10-GM coding. Persons infected with SARS-CoV-2 during 2020 and matched contemporary controls without infection were followed until September 30, 2021, for a minimum of three and a maximum of 18 months using the date of COVID-19 onset as the index date for randomly selected match groups. Persons infected with influenza in the first half of 2018 were followed until September 30, 2019. Following the NICE guidelines on long COVID [9] and the clinical case definition of post-COVID conditions proposed by the World Health Organization (WHO) [10] the post-COVID phase was defined as starting three months after the initial diagnosis of COVID-19. Outpatient services are documented per quarter rather than on a daily basis in the German statutory health care billing system. A diagnosis was therefore associated with the post-COVID period if it was newly documented in the second quarter after the index date or later. This operationalization ensured a time interval of at least three months between the date of COVID-19 diagnosis and post-COVID condition outcome incidence.

### Cohorts

The COVID-19 cohort included individuals with a birthdate before 2003 (aged 18 and older) and a polymerase chain reaction (PCR)-confirmed COVID-19 diagnosis (ICD-10 U07.1) in 2020. To calculate risk exposure time, we defined the index date by using the date of an outpatient PCR test or the date of admission to a hospital with a COVID-19 diagnosis. In rare cases where no PCR test had been billed to the insurance company and no hospital stay was recorded, other documented events, such as the start of sick leave or the first contact with the responsible physician, served to determine the index date. The contemporary control cohort included individuals who were not diagnosed with COVID-19 as ICD-10 U07.1 or ICD-10 U07.2 between January 1, 2020 and September 30th, 2021. The Influenza cohort included persons born before 2001 and an ICD-10 J10 code between January 1, 2018, and June 30, 2018 as this was the last Influenza epidemic in Germany where the dominate Yamagata variant was not covered by the available trivalent vaccine in Germany [11, 12].

We excluded individuals with COVID-19 diagnosis without laboratory virus detection (ICD-10-GM: U07.2) from the COVID-19 cohort and contemporary control cohort to reduce distortions due to misclassification. We further excluded individuals who were not continuously insured with the respective health insurance company between January 1, 2019 and September 30th, 2021 (or death) and for the Influenza cohort between January 1, 2018 and September 30th 2021 (or death) respectively, to ensure that relevant outcomes and preexisting health conditions were visible in our data. For each individual, preexisting medical conditions were assessed for at least 12 months prior to the matching point of the COVID-19 and control cohorts. Starting from the index date assigned from the COVID-19 case matched individuals were jointly followed for a maximum of 18 months. Only patients from the year 2020 for the COVID-19 and contemporary control and 2018 for the Influenza cohort were selected, as this ensured that the effect was not influenced by vaccinations (Vaccination for SARS-CoV-2 in Germany started as of December 27, 2020).

### Ethics and registration

The POINTED study protocol was approved by the ethics committee of the Technische Universität Dresden (IRB00001473 approval number: BO-EK (COVID)-482,102,021) and adheres to all relevant administrative and legal regulations. The study was registered at ClinicalTrials.gov on the October 12, 2021 (NCT number: NCT05074953).

### Data

The underlying data sources were set up for the “Post-COVID-19 Monitoring in Routine Health Insurance Data” (POINTED) consortium [6] to study the long-lasting effects of the COVID-19 pandemic in Germany. The POINTED consortium is coordinated by the Center for Evidence-Based Healthcare (ZEGV) at the TU Dresden and consists of the German National Public Health Authority, the Robert Koch Institute, health research institutes, and statutory health insurances.

We used routine health care data from different German statutory health insurances: Techniker Krankenkasse, BARMER, DAK Gesundheit, IKK classic, AOK PLUS, and several company health insurance funds (InGef [13]). In total, these data cover approximately 39 million individuals, which corresponds to nearly half of the total German population. In addition to sociodemographic characteristics (age and sex) and vital status (via the date of death), we had access to comprehensive information on health care utilization in the outpatient and inpatient health care sectors. The data comprise records on diagnoses (according to the International Statistical Classification of Diseases and Related Health Problems—German Modification, ICD-10-GM), medical procedures (according to the “Operationen- und Prozedurenschluessel,” OPS; German modification of the International Classification of Procedures in Medicine, ICPM), information on outpatient medical services (according to “Einheitlicher Bewertungsmassstab,” EBM), and prescribed medications (according to the German Anatomical Therapeutic Chemical (ATC) Classification).

### Matching

To minimize differences between the COVID-19 and control cohorts in terms of covariates that may confound relationships between outcomes and exposure, we applied 1:3 matching with replacement for COVID-19 to non-COVID-19 contemporary controls and 1:1 for COVID-19 to influenza patients. For each individual in the COVID-19 cohort, we selected three non-COVID-19 individuals with identical age (in years) and sex. Influenza controls were assigned using the same age, sex and disease severity (outpatient, hospital, ICU). We chose exact matching on these characteristics to facilitate stratified analysis. In addition, we accounted for the presence of covariates by propensity score matching. The estimation of the propensity score was based on logistic regression including all insured individuals.

After matching individuals with COVID-19 and controls, we excluded individuals from the match groups if they died before the beginning of the post-COVID phase, i.e., within the quarter of the COVID-19 diagnosis or the following quarter. We also excluded individuals with COVID-19 who lacked a matching partner. When analyzing specific health outcomes, we further excluded individuals from the analysis if the considered outcome was documented in two of the four quarters preceding in the outpatient setting or once in the inpatient setting. To maintain cohort balance on covariates, complete match groups of COVID-19 and control cases were excluded if the outcome was preexisting in the individual with COVID-19 or all of their matched non-COVID-19 contemporary control cases. For estimation, data from individuals in the contemporary control cohort were weighted with the inverse number of individuals remaining in the respective match group (i.e., weights between 1/3 and 1) to ensure that total weights in the control cohort added up to the number of individuals in the COVID-19 cohort. Due to a smaller pool of potential Influenza patients, only a subgroup of COVID-19 patients could be used and influenza patients generally had to be included multiple times in the matching process.

### Outcomes

Although the ICD-10 catalog lists codes for post-viral disease (B94.8) and as of 2021 also for post-COVID condition (U09.9), these codes may largely underestimate the proportion of affected patients [14]. For this reason, we follow the widely used strategy to define post-COVID by symptoms and conditions associated with it. Based on published literature, previous work developing a core outcome set [15], and the clinical expertise of the author team, we selected a large set of 96 outcomes covering multiple organ systems and diagnosis/symptom complexes (Supplemental Table 1). These outcomes constitute new-onset morbidity documented in ICD-10-GM codes by a physician or psychotherapist in the inpatient or outpatient sector within the statutory healthcare system. Of these 96 symptoms and conditions, seven were selected to represent the WHO post-COVID clinical case definition (malaise/exhaustion, chronic fatigue syndrome, dyspnea, respiratory insufficiency, chest pain, cognitive impairment, memory disorder). These symptoms and conditions cover the three main clusters of persistent fatigue as well as respiratory and cognitive problems. Furthermore, four conditions were selected for the less common but potentially more severe tissue damages (pulmonary embolism, lung damage, pericarditis, and myocarditis). Lastly, two negative control endpoints were defined as melanoma and tinea pedis. Both endpoints are assumed not to be caused by a SARS-CoV-2 or Influenza infection, but subject to the same unmeasured exposures such as health seeking behavior (detection bias) after an infection or in case of contemporary controls also to lockdown effects [16].

### Covariates

We used information on preexisting chronic conditions as available health records from 2019 and 2017, respectively to adjust for potential confounders in the relationship of exposure (COVID-19) and endpoints. The approach is the same as in a previous study [6]. For each individual, we used information on preexisting health conditions in the four quarters preceding the index date. The 34 prevalent morbidities were based on published evidence and clinical expertise. In addition, we included age, sex, and the number of recorded inpatient and outpatient contacts as covariates. In line with previous studies [17], we included the severity of COVID-19 as a stratification feature and differentiated between (1) individuals with outpatient diagnoses of COVID-19, (2) individuals with a hospitalization with COVID-19, and (3) individuals requiring intensive care and/or mechanical ventilation (ICU) with COVID-19 or influenza.

### Statistical analyses

The incidence rates (IRs) of the endpoints per 1,000 person-years were estimated. Differences between COVID-19 and non-COVID-19/influenza patients were estimated using Poisson regression models to with incidence rate ratios (IRRs) and corresponding 95% confidence intervals(CI). As a prerequisite, we derived aggregated information on each health outcome by counting incident cases of the respective endpoint within the COVID-19 and control cohorts. Since the number of incident cases for each outcome varied across the match groups of contemporary controls, we assigned weights to the remaining cases that added up to 1. The pooling of individual-level data was not possible due to data protection restrictions. Each authorized institute calculated the required aggregate statistics and provided them to ZEGV, where regressions based on combined aggregate data were performed.

To synthesize evidence across datasets, point estimates from aggregate matched data were found to be the same compared with Poisson regression based on individual level data [18]. The characteristics of Poisson regression applied to aggregate count data allowed for consistent estimation of incidence rates regardless of the distribution of the outcome on the individual level when the conditional mean function is correctly specified [19]. While the variance estimates for a 1:1 matching are the same model estimates on individual as well as aggregate data level, the variance estimates from aggregates for 1: M matching tend to be larger, meaning that the statistical significance of the presented effects may be underestimated. However, in the case of a 1:1 matching with replacement from a comparatively small pool of Influenza controls the variance is larger in the models based on aggregate data. To address this issue, simulations were conducted and it was determined that weighting by natural persons per control case provided appropriate variance estimates. Utilizing a main advantage of Poisson regression, we adjusted for differences in times at risk (time between the index date and the end of the observation period or death) due to inclusion of these times as offset in the model. Stratified aggregation enabled us to deploy separate estimators for age, sex, and severity of the infection.

To investigate the persistence of the endpoints we employed the Kaplan–Meier estimator [20]. This estimator allowed to approximate the course of the symptoms and conditions under each censoring due to death or end of observation time for the 6 quarters after the index date. The absence of the diagnosis was interpreted as a loss of the symptom or condition. All analyses used the statistical programming language R version 3.6.3 [21].

## Results

### Selection of the study population

**Fig. 1:**
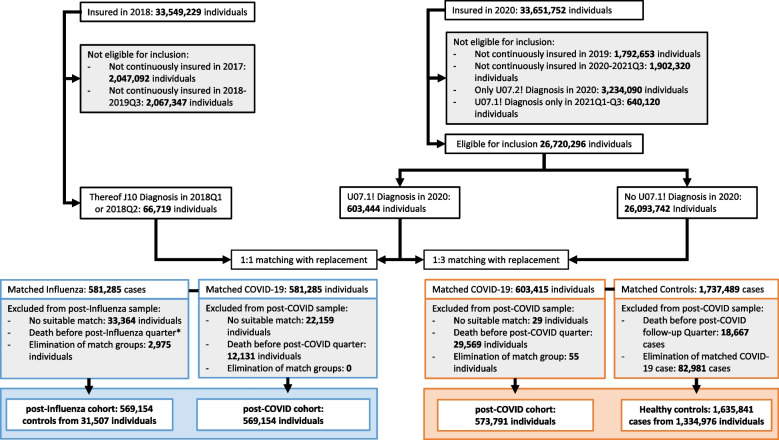
Flowchart selection of the COVID-19, Influenza and contemporary control cohort. *Persons with Influenza who died before the first post-Influenza quarter were eliminated before matching. They are listed under not continuously insured in 2018-2019Q3

From the 33.6 million adult individuals insured with one of the participating insurance companies for at least one day in 2020 we excluded persons not continuously enrolled in 2019 (*n* = 1.8 million) or between January 1, 2020, and September 30, 2021 (*n* = 1.9 million), persons with a COVID-19 diagnosis without laboratory confirmation (ICD-10 U07.2) (*n* = 3.2 million), and persons with a COVID-19 diagnosis in the first three quarters of 2021 (*n* = 0.6 million) (Fig. 1). From the remaining sample, 603,415 individuals with a COVID-19 diagnosis were matched 1:3 to contemporary controls without COVID-19 in the observational period. After mutual exclusion of cases in the COVID-19 and control cohort, who were not observable for at least two quarters after index the final study population consisted of 573,791 individuals with COVID-19 and 1,334,976 non-COVID-19 individuals serving as controls in 1,635,841 cases. Following the same approach, the comparison between COVID-19 and influenza cohorts was based on 569,154 adults with COVID-19 in 2020 and the same number of influenza controls who were resampled from 33,364 individuals with documented influenza in 2018. 22,159 cases were lost in the COVID-19 cohort due to lack of suitable Influenza controls from the year 2018 (Fig. 1).

### Description of the study population

Among the 573,791 individuals included with PCR-test confirmed COVID-19 diagnosis in 2020, more than half were female (58.3%), one third were aged between 18 and 39 years (34.2%) and 7.8% above the age of 80 years. Both control cohorts showed the same sex and age specific characteristics due to exact matching on these properties. Regarding the course of the acute COVID-19 disease, 6.8% of individuals (*n* = 39,181) were hospitalized, and 1.7% (*n* = 9,529) received intensive care treatment and/or mechanical ventilation (Table 1). At least one out of seven reported post-COVID symptoms and conditions as defined by the WHO clinical case definition among adults was present in 5.2% in the COVID-19 cohort, 2.4% in the contemporary control cohort and 2.9% in the influenza cohort. The most common WHO post-COVID symptoms and conditions were malaise/exhaustion and dyspnea, each affected 2.2% of the COVID-19 cohort. Among four reported conditions involving tissue damage, conditions most commonly observed in the COVID-19 cohort were pulmonary embolism with 1,228 cases (0.2%), lung damage with 729 cases (0.1%), pericarditis with 144 cases and myocarditis with 107 cases (Table 2). The distribution of the population size in different quarters are documented in Supplemental Table 2.

**Table 1:** Distribution of socio-demographic characteristics and severity of the infection of the COVID-19, contemporary control, and influenza cohort after matching. The corresponding COVID-19 cohort, which was used in comparison with the influenza cohort is smaller as not all COVID-19 patients could be matched with influenza controls

**Domain**	Category	*N* (%) COVID-19 in 2020 (*n*=573,791	*N* (%) Contemporary controls no COVID-19 (*n*=1,635,841)	*N* (%) Influenza in 2018 (*n*=569,154)
**Age**	18-29	111,401 (19.4)	309,488 (18.9)	95,505 (16.8)
	30-39	84,939 (14.8)	236,547 (14.5)	99,886 (17.6)
	40-49	100,746 (17.6)	289,485 (17.7)	100,222 (17.6)
	50-59	125,819 (21.9)	365,689 (22.4)	125,070 (22.0)
	60-69	68,386 (11.9)	199,199 (12.2)	68,787 (12.1)
	70-79	37,519 (6.5)	109,683 (6.7)	37,250 (6.5)
	80-89	34,950 (6.1)	99,292 (6.1)	34,461 (6.1)
	90+	10,031 (1.8)	26,458 (1.6)	7,973 (1.4)
**Sex**	Male	239,576 (41.8)	682,474 (41.7)	237,133 (41.7)
	Female	334,215 (58.3)	953,367 (58.3)	332,021 (58.3)
**Severity of Infection**	Outpatient	525,081 (91.5)	-	521,813 (91.7)
	Hospital	39,181 (6.8)	-	38,951 (6.8)
	ICU	9,529 (1.7)	-	8,390 (1.5)

**Table 2:** Distribution of main health outcomes the COVID-19, contemporary control, and influenza cohort after matching. Depending on endpoints, the risk population changes as preexisting diagnoses were excluded in the analysis

**Domain**	Category	*N* (%) COVID-19 in 2020	*N* (%) Contemporary controls no COVID-19	*N* (%) Influenza in 2018
**WHO post-COVID**	Any symptom or condition	29,660 (5.17)	38,875 (2.38)	16,264 (2.86)
	Malaise/exhaustion	11,555 (2.15)	15,958 (1.10)	6,705 (1.35)
	Chronic fatigue syndrome	2,115 (0.37)	1,073 (0.07)	270 (0.05)
	Dyspnea	11,880 (2.17)	10,832 (0.72)	4,409 (0.85)
	Respiratory insufficiency	3,338 (0.59)	4,276 (0.27)	1,917 (0.35)
	Chest pain	203 (0.04)	249 (0.02)	53 (0.01)
	Cognitive Impairment	2,603 (0.46)	5,048 (0.32)	2,118 (0.39)
	Memory disorder	729 (0.13)	2,660 (0.17)	735 (0.13)
**Tissue damage**	Pulmonary embolism	1,228 (0.22)	1,070 (0.07)	485 (0.09)
	Lung damage	729 (0.13)	639 (0.04)	353 (0.06)
	Pericarditis	144 (0.03)	106 (0.01)	80 (0.01)
	Myocarditis	107 (0.02)	73 (0.00)	48 (0.01)

### Incidence of post-COVID symptoms and conditions

**Fig. 2:**
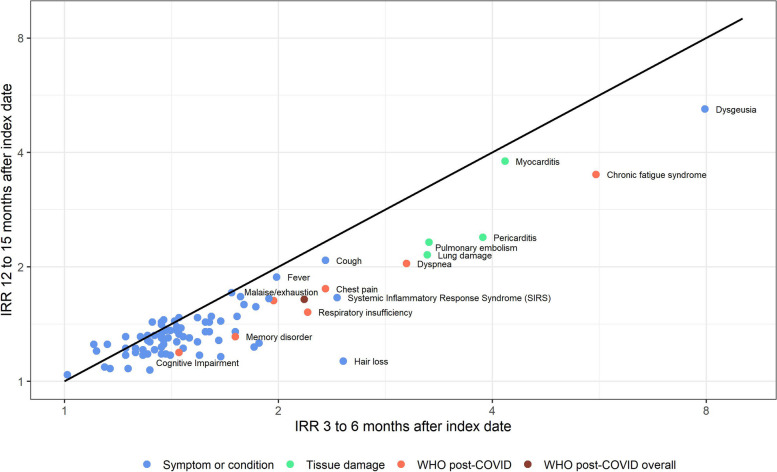
Scatter plot of incidence risk ratios of a Poisson regression of COVID-19 compared to the matched contemporary control cohort 3 to 6 and 12 to 15 months after infection on logarithmic scale

Figure 2 displays a scatter plot of incidence rate ratios (IRR) for selected health outcomes (Supplemental Table 1) comparing the COVID-19 to the contemporary control cohort at 3–6 months and 12–15 months after the index date. Health outcomes were selected if the incidence rate (IR) per 1,000 person-years was above 0.5% in 3–6 months after infection in the COVID-19 cohort.

The highest relative risk was observed for dysgeusia/anosmia with an IRR of 7.9 which decreased to 5.4 after 12 to 15 months. In general, most symptoms and conditions diminished over time on the population level. Common unspecific symptoms like fever and cough showed similar estimates over the observed quarters. Except for myocarditis, the IRRs for conditions involving tissue damage decreased over time, yet remained elevated. A reduction in relative risk over time was seen for all WHO post-COVID symptoms and conditions. Nevertheless, the estimates were much higher for chronic fatigue syndrome (CFS) or dyspnea compared to cognitive impairment or memory loss. The overall risk for any incident post-COVID symptom or condition decreased from 2.18 (95%CI: 2.13–2.22) to 1.64 (95%CI: 1.58–1.71). As can be seen from the IR difference this estimate mostly depends on malaise/exhaustion and dyspnea (Table 3).

**Table 3:** Estimates of Poisson regression of COVID-19 compared to the matched contemporary control and influenza cohort 3 to 6 and 12 to 15 months after infection

COVID-19 versus Non-COVID						
	3 to 6 months after index date			12 to 15 months after index date		
**Symptom or Condition**	IRR	95% CI	IR difference	IRR	95% CI	IR difference
**WHO post-COVID**						
WHO post-COVID definition	2.18	2.13–2.22	75.95	1.64	1.58–1.71	47.57
Malaise/exhaustion	1.97	1.91–2.03	28.82	1.63	1.54–1.73	21.89
Chronic fatigue syndrome	5.60	5.02–6.25	8.26	3.50	2.92–4.19	6.77
Dyspnea	3.03	2.92–3.14	39.45	2.04	1.90–2.19	26.47
Respiratory insufficiency	2.20	2.07–2.34	8.77	1.51	1.34–1.72	3.97
Chest pain	4.09	3.02–5.54	0.55	2.64	1.55–4.50	0.37
Cognitive Impairment	1.45	1.36–1.54	3.91	1.19	1.06–1.33	1.80
Memory disorder	1.74	1.61–1.89	3.35	1.31	1.12–1.52	1.60
**Tissue damage**						
Pulmonary embolism	3.26	2.90–3.65	4.05	2.32	1.89–2.85	3.08
Lung damage	3.24	2.79–3.77	2.39	2.15	1.65–2.79	1.69
Pericarditis	3.88	2.70–5.56	0.51	2.39	1.34–4.25	0.41
Myocarditis	4.17	2.71–6.42	0.39	3.79	1.85–7.75	0.48
**Control endpoints**						
Melanoma	1.24	1.06–1.44	0.34	0.92	0.71–1.18	-0.18
Tinea pedis	1.24	1.13–1.36	0.87	1.24	1.04–1.48	0.98
**COVID-19 versus influenza**						
	3 to 6 months after index date			12 to 15 months after index date		
**Symptom or Condition**	IRR	95% CI	IR difference	IRR	95% CI	IR difference
**WHO post-COVID**						
WHO post-COVID Definition	1.87	1.75–2.01	63.37	1.37	1.27–1.48	31.15
Malaise/exhaustion	1.66	1.50–1.83	22.53	1.49	1.31–1.68	17.57
Chronic fatigue syndrome	8.23	4.90–13.81	8.77	1.20	0.92–1.55	1.53
Dyspnea	2.65	2.33–3.02	35.68	1.58	1.36–1.83	15.29
Respiratory insufficiency	1.64	1.34–2.00	5.59	1.52	1.16–2.00	3.77
Chest pain	4.09	1.27–13.23	0.73	2.64	0.76–9.18	0.52
Cognitive Impairment	1.18	0.98–1.43	1.77	1.13	0.89–1.43	1.20
Memory disorder	2.27	1.65–3.12	4.24	1.19	0.87–1.62	1.06
**Tissue damage**						
Pulmonary embolism	2.58	1.74–3.81	3.45	1.96	1.27–3.03	2.62
Lung damage	2.11	1.33–3.34	1.76	2.46	1.30–4.67	1.80
Pericarditis	1.92	0.73–5.03	0.33	2.50	0.67–9.37	0.43
Myocarditis	2.42	0.70–8.34	0.30	3.47	0.68–17.68	0.45
**Negative control endpoints**						
Melanoma	0.61	0.43–0.87	-1.11	0.64	0.41–0.99	-1.10
Tinea pedis	1.13	0.84–1.52	0.50	0.89	0.65–1.22	-0.62

*IRR* Incidence rate ratios, *IR* Incidence difference per 1000 person-years

When compared to the influenza cohort the estimated IRR for post-COVID condition was 1.87 (95% 1.75–2.01) and 1.37 (95%1.27–1.48) at 3–6 and 12–15 months after index date. Except for CFS at 3–6 months after the index date, IRRs for individual post-COVID condition were generally smaller when comparing the COVID-19 cohort to the influenza cohort than to the contemporary control cohort. However, the IRR for conditions involving tissue damage was similar between the COVID-19 cohort compared to the influenza cohort and compared to the contemporary control cohort. Both control endpoints for COVID-19 vs. contemporary controls showed an IRR of 1.24 in the 3 to 6 months after index date (Table 3).

Overall, the IR difference between the COVID-19 cohort and contemporary control cohort for post-COVID symptoms and conditions was 75.95 per 1000 person-years at 3 to 6 months after index date and 47.57 after 12 to 15 months after index date (Table 3). Among subgroups, post-COVID condition was more pronounced among women with an IR difference of 81.11 than among men with an IR difference of 68.90 per 1,000 person-years 3 to 6 months after index date. These values decreased to 51.16 and 42.18, respectively in the 12 to 15 months after index date. However, IRR estimates were not significantly different between the sexes. Sex specific differences were most pronounced for CFS, which had an IRR of 6.71 and 4.28 for men and 5.24 and 3.26 for women in both periods. In contrast, for tissue damage the IRR among men was higher for pulmonary embolism and lung damage, but lower for pericarditis and myocarditis (Supplemental Tables 3 and 4).

The IRR for the outcomes increased with the severity of COVID-19. In the outpatient sector, the overall relative risk for WHO post-COVID symptoms and conditions amounted to 2.04 after 3 to 6 months after index date and 1.57 after 12 to 15 months after index date compared to contemporary controls. Considering COVID-19 cases treated in hospital the IRR was 2.62 and 2.01 in the respective time periods and for individuals in ICU 4.40 and 3.44. Comparing COVID-19 and influenza in the 3 to 6 months after index date the IRRs were 1.93 for outpatient, 1.56 for hospital, and 1.80 for ICU patients. IRRs decreased to 1.39 for outpatient, 1.13 for hospital, and 1.15 for ICU patients after 12 to 15 months. The IR difference was highest for the ICU patients with 400 and 304 after 3 to 6 and 12 to 15 months after index date compared to contemporary controls and 231 respectively 55 compared to influenza. The IRR for WHO post-COVID condition were lower in comparison to the influenza cohort than to the contemporary control cohort. For hospitalized and ICU patients the IRR for the COVID-19 cohort was much higher compared to contemporary controls without a hospital stay. For the COVID-19 cohort the IRR compared to the influenza cohort was similar between outpatient and hospitalized sector. (Supplemental Tables 5, 6 and 7). Considering age specific differences, we found the highest absolute risk difference of post-COVID condition for persons aged 60–69 to 80–89 (IR = 95.33 to 99.55) and the highest relative risk for age groups 40–69 (IRR = 2.61). The highest IRR for dyspnea was observed in the age group 40–49 years and for malaise/exhaustion in the group 60–69 years (Supplemental Table 8).

### Persistence of post-COVID symptoms and conditions

**Fig. 3:**
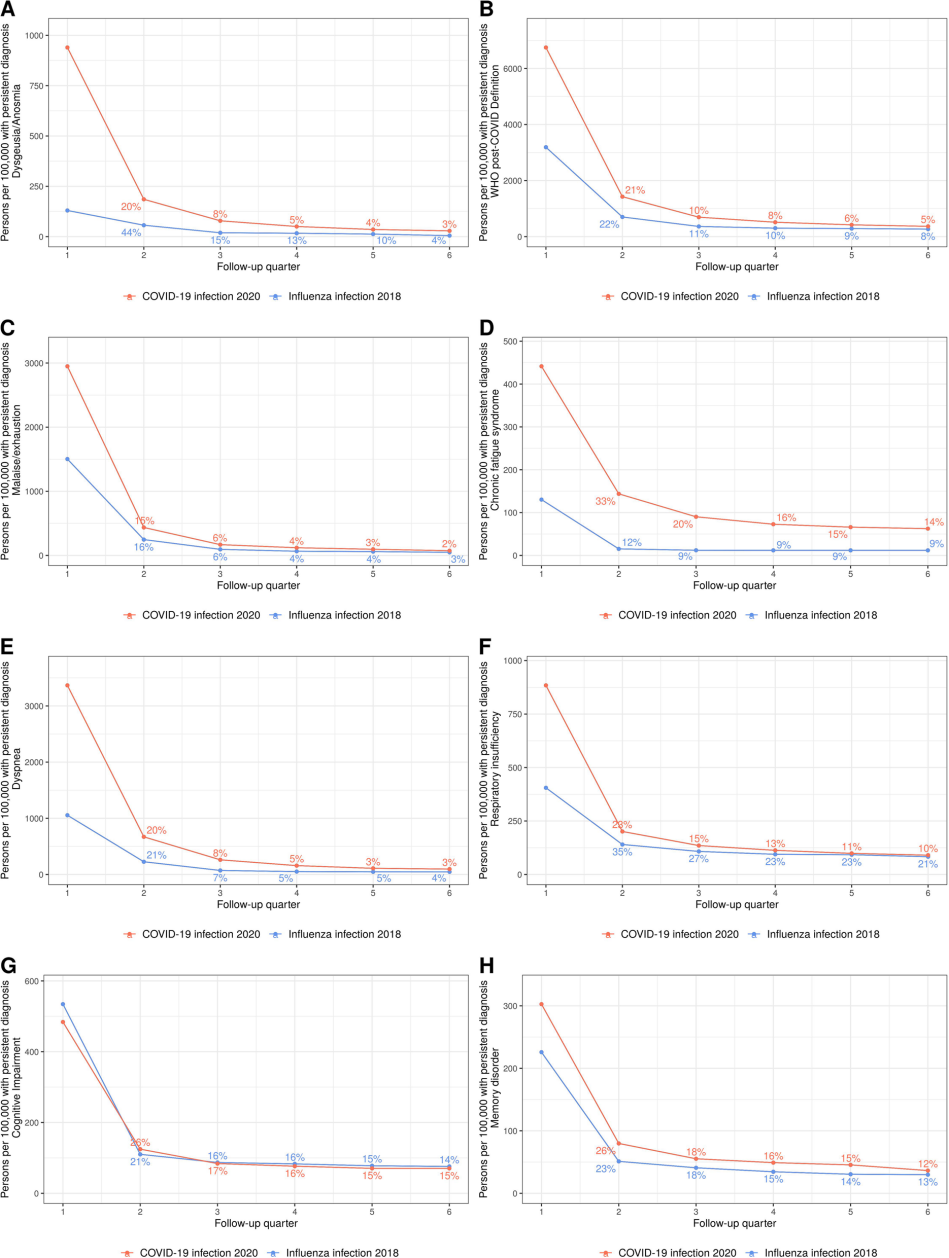
Persistence of symptoms and conditions over 6 quarters for A Dysgeusia/Anosmia (ICD-10 R43.0,R43.2,R43.8), B WHO post-COVID condition Definition, C Malaise/exhaustion (ICD-10:R53), D Chronic fatigue syndrome (ICD-10:G93.3), E Dyspnea (ICD-10:R06.0, R06.2, R06.88), F Respiratory insufficient (ICD-10:J96), G Cognitive Impairment (ICD-10:F06.7, U51.1, U51.2) and H Memory disorder (ICD-10:R41). Chest pain (ICd-10 R07.1) was not persistent during the time span

To gain insight into the persistence of diagnoses at the individual level we identified persons with symptom onset in the first three months after index and followed them over time. Figure 3 shows the persistence of symptoms in the COVID-19 cohort and the influenza cohort up to quarter 6 (15 to 18 months) following infection. Dysgeusia/anosmia was a common symptom related to a SARS-CoV-2 infection. In the COVID-19 cohort, about 1,000 persons per 100,000 person-years had this diagnosis coded in quarter 1, but for 80% of these persons the diagnosis was no longer coded in quarter 2. In the influenza cohort, 130 persons per 100,000 person-years were affected in quarter 1 and 56% lost the diagnosis by quarter 2. In quarter 6, only 3 to 4% in both cohorts had the diagnosis (Fig. 3 A). At least one symptom related to post-COVID condition according to the 2021 WHO consensus was diagnosed in 6,747 per 100,000 persons in the COVID-19 and 3,189 per 100.000 in the influenza cohort. The rate of persons diagnosed with any of these symptoms was reduced by 90% in quarter 3 and only 5 to 8% were affected in quarter 6. Within the WHO defined post-COVID symptoms and conditions, persistence rates were similar, whereas the initial rate was higher in the COVID-19 cohort. The most persistent symptoms in the COVID-19 cohort were memory disorder (12% still affected in quarter 6), cognitive impairment (15% still affected) and CFS (14% still affected). From the individuals with post-COVID symptoms and conditions according to the WHO definition in the first quarter after infection (6.75%), 5.4% remained affected in quarter 6 (Fig. 3B). This means that 0.36% of adults with COVID-19 related symptoms 0–3 months after SARS-CoV-2 infection were still suffering from it after 18–21 months. The corresponding estimate for post-influenza was 0.27%.

## Discussion

### Main results

Post-acute infectious syndromes can manifest in several ways from tissue damage to the onset of autoimmunity, dysregulation of the microbiota–gut–brain axis and symptoms resulting from the chronic stimulation of the immune system [1]. The difference for the WHO post-COVID condition definition between the COVID-19 cohort in 2020 and the Influenza cohort in 2018 was smaller than between COVID-19 and contemporary controls. The IRR for WHO post-COVID condition was highest in the middle age groups between 40 and 69 years. While the IRR for post-COVID symptoms and conditions decreased in the following quarters after infection on the *population level*, the decline in the persistence of these symptoms and conditions on the *individual level* was larger. In addition, the persistence was similar between the COVID-19 and influenza cohort.

Some individuals might be more susceptible to post-viral symptoms and conditions and also experience a relapse of symptoms after initial recovery. Preexisting frailty was also associated with a higher likelihood for post-COVID condition [22]. Factors associated with prolonged time to symptom-free were increased age, female sex, obesity, smoking, respiratory disease, depression, multimorbidity, lower socioeconomic status, hospitalization with COVID-19, and being a healthcare worker [5, 22–24]. The lower risk of post-COVID condition in individuals above the age of 70 might be due to the high case fatality rate in this age group [25]. In line with our findings, a previous cohort study with routine healthcare data found a decreasing risk for post-COVID condition after the age of 60 years [26].

Although it has been shown that vaccination and antiviral therapies during the acute phase of COVID-19 decrease the risk of post-COVID condition, no therapies in the post-COVID phase are currently approved [22, 27, 28]. However, several trials were conducted and are ongoing for pharmaceutical as well as non-pharmaceutical treatments like hyperbaric oxygen therapy [2, 29].

### Comparison with other cohort studies

At 3 to 6 months after index date 21% in the COVID-19 cohort and 22% in the influenza cohort still suffered from post-COVID conditions according to the WHO definition. A community survey in the UK among adults with confirmed or suspected SARS-CoV-2 infection found at least one symptom to persist for three months for 30% of men and 40% of women [26]. Another survey cohort reported persistent symptoms for 23% of the individuals with confirmed SARS-CoV-2 infection after six months and 17% after 24 months [30]. A cohort study of patients hospitalized due to COVID-19 reported post-COVID symptoms for 68% after 6 months and 55% after 24 months [31]. The divergence in study results might be due to selective participation of those still suffering from symptoms and the severity of some symptoms reaching subclinical levels. Three previous cohort studies of hospitalized patients found that post-viral symptoms were more severe for COVID-19 compared to influenza [14, 32, 33]. A review of studies on the persistence of smell or taste disorders following COVID-19 suggests that 96% of affected patients recovered their sense of smell and 98% their sense of taste after 6 months [34].

The IRR for pulmonary embolism observed in the present study was similar in another matched cohort based on data from national health registries in Sweden [35]. The IRRs for myocarditis and pericarditis were also in line with the published literature on COVID-19 and much higher than the reported relative risk from mRNA vaccines against SARS-CoV-2 [36]. In our study, the effect was similar regardless which control cohort was considered. This suggests the property of tissue damage is more specific for COVID-19 than for influenza.

### Strengths and limitations

The main strength of this analysis is its large dataset including over half a million COVID-19 patients and corresponding contemporary controls as well as historical control form the 2018 Influenza epidemic with up to 18 months follow-up. This unselected sample from all over Germany covers both outpatient and inpatient care and thus, constitutes a unique and comprehensive source of evidence. Our analysis is based on confirmed diagnoses documented by ambulatory physicians/psychotherapists and hospital discharge diagnosis. Accordingly, our results are not subject to possible distortions resulting from selective, incomplete, or inadequate self-reporting of symptoms. To avoid confounding in the relationships between outcomes and exposure, we applied matching on relevant covariates, age, sex, where possible disease severity, several prevalent diseases, as well as utilization of outpatient and inpatient care. The results were confirmed by the use of control endpoints.

We observed a protective effect of COVID-19 versus Influenza but not versus contemporary controls considering melanoma. This may be attributed to a reduction of cancer screening during lockdown. The IRR for CFS 3 to 6 months after index date in the COVID-19 cohort compared to the Influenza cohort was much higher than compared to the contemporary control cohort (Table 3). This effect might be caused by a higher awareness of the CFS diagnosis during the pandemic. Restricting the analysis only to those cases with an infection in the first quarter of 2020 did result in an IRR of 4.42 (95%CI 2.13–9.18) COVID-19 to contemporary controls and 1.82 (95%CI 1.23–2.70) COVID-19 to Influenza. As the persistence of CFS decreases over time, it can be debated that during the pandemic, suggesting more severe exhaustion was coded under CFS and the “real” CFS cases constitute a minority in this patient group.

We cannot exclude that our results were affected by unmeasured confounding, although we minimized differences between the COVID-19 and control cohorts by matching. Vaccination status could not be validly assessed in German claims data. Individuals with a mild or asymptomatic course of COVID-19 were likely to be underrepresented in our study because SARS-CoV-2 infections may not have been documented [37], especially in the first months of the pandemic. In addition, individuals with undocumented SARS-CoV-2 infection may have been included in the control cohort. However, this bias should be small in the first, two waves of the pandemic in Germany. In October 2021, the third wave hit Germany. This, together with the appearance of the Omicron variant in January 2022, has increased the size of the COVID-19 cohort to such an extent that the contemporary control cohort is contaminated for further analyses. Furthermore, later virus variants have been found to induce fewer post-COVID condition cases, which would reduce the estimates [24].

## Conclusion

Post-COVID condition can manifest in several ways. Two of them, namely tissue damage and symptoms and conditions because of chronic stimulation of the immune system were investigated here for adults. The effect of COVID-19 for tissue damage was similar in comparison to both control cohorts. The chronic stimulation of the immune system was used to create a post-COVID condition definition by the WHO, which included fatigue, respiratory as well as cognitive problems. The difference between the COVID-19 cohort in 2020 and the influenza cohort in 2018 was smaller than between COVID-19 and contemporary controls. The main contributions in terms of absolute excess risk came from dyspnea and malaise/exhaustion, while the highest IRR was observed for chronic fatigue syndrome. After one year, only a minority of the initial patients still suffer from post-COVID condition with a similar pattern of persistence among patients with influenza. However, given the increasingly endemic nature of the disease people will be infected every season, resulting in a constant patient population that needs care.

## Supplementary Information

Supplementary Material 1. 

## Data Availability

The raw data used in this study cannot be made available in the manuscript, the supplemental files, or in a public repository due to German data protection laws (Bundesdatenschutzgesetz). The aggregated data is stored on a secure drive at ZEGV. The R code of the analysis can be made available upon request by the corresponding author.

## Abbreviations

CI: Confidence interval
CFS: Chronic fatigue syndrome
ICD-10-GM: International Statistical Classification of Diseases and Related Health Problems, 10th revision German modification
ICU: Intensive care and/or mechanical ventilation
IR: Incidence rates per 1,000 person-years
IRR: Incidence rate ratio Ras effect estimates in a Poisson regression model
PCR: Polymerase chain reaction
WHO: World Health Organization
