# Supplementary material for: Post-viral symptoms and conditions are more frequent in COVID-19 than influenza, but not more persistent
Source: BMC Infect Dis. 2024 Oct 9;24:1126. doi: 10.1186/s12879-024-10059-y (PMC11465902; doi:10.1186/s12879-024-10059-y)
Supplement: Supplementary file 1 — Supplementary Material 1. [file 12879_2024_10059_MOESM1_ESM.docx]

**Supplement**

Table 1 Chosen Endpoints in the study

| **Symptom or Condition** | ICD-10 Code |
| --- | --- |
| **WHO post-COVID** |  |
| Malaise/exhaustion | R53 |
| Chronic fatigue syndrome | G93.3 |
| Dyspnea | R06.0, R06.2, R06.88 |
| Respiratory insufficiency | J96 |
| Chest pain (when breathing) | R07.1 |
| Cognitive impairment | F06.7, U51 |
| Memory disorder | R41 |
| **Tissue damage** |  |
| Pulmonary embolism | I26 |
| Lung damage | J84.0, J84.1, J93.8, J98.4 |
| Pericarditis | I30.0, I30.8, I30.9, I31.9 |
| Myocarditis | I40 |
| **Control endpoints** |  |
| Melanoma | C43 |
| Tinea pedis | B35.3 |
| **Other Endpoints** |  |
| Abdominal pain | R10.1, R10.2, R10.3, R10.4 |
| Acute pain | R52.0 |
| Adjustment disorder | F43 |
| Anuria, oliguria | R34 |
| Anxiety disorder | F40, F41 |
| Arthritis | M02, M13 |
| Ascites | R18 |
| Behavioral symptoms | R46 |
| Cachexia | R64 |
| Carditis due to viruses | B33.2 |
| Changes in bowel habits | R19.4 |
| Concentration impairment/Concentration  Deficit | R41.8 |
| Cough | R05 |
| COVID toe | L93.2 |
| Depression | F32, F33, F34.1 |
| Developmental delay | F80, F81, F82, F83, F84, F88, F89 |
| Diarrhea | A09, A58, K52.9, R19.4, R19.5 |
| Disorientation | R41.0 |
| Dysgeusia/Anosmia | R43.0, R43.2, R43.8 |
| Dyslexia | R48 |
| Dysmenorrhea | N91, N92, N94 |
| Dysphagia | R13 |
| Dysuria | R30 |
| Emotional and behavioral disorder | F54, F59, F91, F92, F93, F95, F98.8, F98.9 |
| Epistaxis | R04.0 |
| Eye pain | H57.1 |
| Facial nerve paralysis | G51 |
| Fever | R50 |
| Flatulence | R14 |
| Gangraen | R02 |
| General symptoms | R68 |
| Hair loss | L63.9, L65.8, L65.9 |
| Headache | R51 |
| Hearing loss/tinnitus | H91.9, H93.1, H93.2 |
| Heart failure | I11.0, I13.0, I13.2, I50 |
| Heart murmurs | R01 |
| Heartburn | R12 |
| Hemorrhage | R58 |
| Hepatomegaly and splenomegaly | R16 |
| Hoarseness | R49 |
| Hyperhidrosis | R61 |
| Hypotension | I95.0, I95.1, I95.8, I95.9 |
| Impaired balance | R29.6 |
| Joint pain | M25.4, M25.5, M25.8, M25.9 |
| Loss of appetite, eating disorders | F50, R63.0, R63.4, R63.5 |
| Lymphadenopathy | R59 |
| Meningism | R29.1 |
| Mood disorder | R45 |
| Movement disorders | F90, G25.0, G25.2, G25.5, G25.8, R25, R26 |
| Multisystemic inflammatory syndrome | D76.4, M08.2, M30.3, R65, U10.9 |
| Myalgia | M79.1, M79.2, M79.6 |
| Myocardial infarction | I20, I21, I22, I23, I24, I25 |
| Nausea | R11 |
| Neurasthenia | F48.0 |
| Neurological manifestation of Post-COVID | G57.3, G61.0, G72.80 |
| Obsessive-compulsive disorder | F42 |
| Oedema | R60 |
| Other cardiac arrhythmias | I44, I45, I46, I47, I48, I49.1, I49.2, I49.3, I49.5, I49.8, I49.9 |
| Other coordination disorders/ataxia | R27 |
| Other symptoms of the urinary system | R39 |
| Pain, not elsewhere classified | R52 |
| Paresis | G82.00, G82.02, G82.35, G83.0, G83.1, G83.2, G83.3, G83.6, G83.8, G83.9 |
| Paresthesia of skin | R20 |
| Pathological findings from male genital  tract | R86 |
| Pathological reflexes | R29.2 |
| Polyuria | R35 |
| Rash | R21, R23 |
| Seizures | G40, R56 |
| Sensation and perception disorder | R44 |
| Shock | R57 |
| Sinus vein thrombosis | I67.6 |
| Sleep disorders | F51, G47.0, G47.1, G47.2, G47.8, G47.9 |
| Somatization disorder | F45 |
| Somnolence, sopor, coma | R40 |
| Speech and language disorders | R47 |
| Stroke | I60, I61, I62, I63, I64, I65, I66, I67, I68, I69 |
| Subcutaneous nodules | R22 |
| Syncope | R55 |
| Tachycardia/Palpitation | I49.0, I49.4, R00 |
| Tetany | R29.0 |
| Thrombosis | I80 |
| Urethral discharge | R36 |
| Urinary retention | R33 |
| Vertigo | H81,H82,R42 |
| Visual disturbances | H53 |

Table2 Follow-up time for each cohort

| **Variable** | Category | N COVID-19 in 2020 | Contemporary controls no COVID-19 until 30/9/2021 | Influenza in 2018 |
| --- | --- | --- | --- | --- |
| **Follow-up time** | 1 Quarter | 573,791 | 1,635,841 | 569,154 |
|  | 2 Quarters | 573,791 | 1,635,841 | 569,154 |
|  | 3 Quarters | 569,067 | 1,613,348 | 563,721 |
|  | 4 Quarters | 278,406 | 792,378 | 276,851 |
|  | 5 Quarters | 149,157 | 421,856 | 147,751 |
|  | 6 Quarters | 67,785 | 192,838 | 66,896 |

Table3 Estimates of Poisson regression of COVID-19 compared to the matched Non-COVID contemporary control 3 to 6 and 12 to 15 months after infection for each sex. IRR – Incidence rate ratios, IR – Incidence difference per 1000 person-years.

| **COVID-19 versus Non-COVID Female** | | | | | | |
| --- | --- | --- | --- | --- | --- | --- |
|  | 3 to 6 months after index date | | | 12 to 15 months after index date | | |
| **Symptom or Condition** | IRR | 95% CI | IR difference | IRR | 95% CI | IR difference |
| **WHO post-COVID** |  |  |  |  |  |  |
| WHO post-COVID Definition | 2.14 | 2.09-2.20 | 81.11 | 1.65 | 1.57-1.73 | 51.16 |
| Malaise/exhaustion | 1.92 | 1.85- 2.00 | 25.21 | 1.64 | 1.52-1.76 | 25.21 |
| Chronic fatigue syndrome | 5.24 | 4.61-5.94 | 9.84 | 3.26 | 2.64-4.01 | 7.80 |
| Dyspnea | 3.12 | 2.98-3.27 | 43.30 | 2.11 | 1.93-2.31 | 24.41 |
| Respiratory insufficiency | 2.01 | 1.85-2.18 | 6.98 | 1.48 | 1.25-1.75 | 3.26 |
| Chest pain | 2.43 | 1.74-3.39 | 0.56 | 1.66 | 0.94- 2.95 | 0.37 |
| Cognitive Impairment | 1.42 | 1.31-1.53 | 3.94 | 1.18 | 1.02-1.36 | 1.77 |
| Memory disorder | 1.75 | 1.58-1.94 | 3.66 | 1.23 | 1.02- 1.50 | 1.30 |
| **Tissue damage** |  |  |  |  |  |  |
| Pulmonary embolism | 2.94 | 2.51-3.43 | 3.36 | 2.17 | 1.66-2.84 | 2.73 |
| Lung damage | 2.66 | 2.16-3.29 | 1.61 | 1.84 | 1.25-2.71 | 1.01 |
| Pericarditis | 5.22 | 3.14-8.66 | 0.61 | 3.84 | 1.79-8.23 | 0.71 |
| Myocarditis | 5.34 | 2.76-10.32 | 0.37 | 5.22 | 1.75-15.56 | 0.48 |
| **Control endpoints** |  |  |  |  |  |  |
| Melanoma | 1.19 | 0.98-1.44 | 0.29 | 0.95 | 0.69-1.30 | -0.12 |
| Tinea pedis | 1.22 | 1.06-1.40 | 0.66 | 1.18 | 0.92-1.52 | 0.62 |
| **COVID-19 versus Non-COVID Male** | | | | | | |
|  | 3 to 6 months after index date | | | 12 to 15 months after index date | | |
| **Symptom or Condition** | IRR | 95% CI | IR difference | IRR | 95% CI | IR difference |
| **WHO post-COVID** |  |  |  |  |  |  |
| WHO post-COVID Definition | 2.24 | 2.16-2.31 | 68.90 | 1.64 | 1.53-1.75 | 42.18 |
| Malaise/exhaustion | 2.06 | 1.95-2.18 | 23.82 | 1.63 | 1.46-1.81 | 17.11 |
| Chronic fatigue syndrome | 6.71 | 5.40-8.34 | 6.09 | 4.28 | 2.95-6.21 | 5.15 |
| Dyspnea | 2.88 | 2.72-3.05 | 34.19 | 1.94 | 1.72-2.17 | 19.24 |
| Respiratory insufficiency | 2.44 | 2.23-2.67 | 11.32 | 1.56 | 1.30-1.87 | 5.07 |
| Chest pain | 2.19 | 1.50-3.20 | 0.53 | 1.92 | 0.85- 4.34 | 0.37 |
| Cognitive Impairment | 1.50 | 1.36-1.66 | 3.87 | 1.21 | 1.00-1.46 | 1.85 |
| Memory disorder | 1.74 | 1.52-1.98 | 2.94 | 1.44 | 1.12-1.85 | 2.07 |
| **Tissue damage** |  |  |  |  |  |  |
| Pulmonary embolism | 3.67 | 3.09-4.37 | 5.03 | 2.54 | 1.84-3.51 | 3.61 |
| Lung damage | 3.89 | 3.15-4.82 | 3.50 | 2.44 | 1.70-3.51 | 2.73 |
| Pericarditis | 2.64 | 1.56-4.46 | 0.36 | 0.88 | 0.32-2.41 | -0.05 |
| Myocarditis | 3.37 | 1.90-5.97 | 0.41 | 2.82 | 1.08-7.36 | 0.47 |
| **Negative control endpoints** |  |  |  |  |  |  |
| Melanoma | 1.33 | 1.04-1.71 | 0.42 | 0.87 | 0.57-1.34 | -0.26 |
| Tinea pedis | 1.26 | 1.10-1.44 | 1.17 | 1.29 | 1.01-1.66 | 1.49 |

Table4 Estimates of Poisson regression of COVID-19 compared to the matched influenza cohort 3 to 6 and 12 to 15 months after infection for each sex. IRR – Incidence rate ratios, IR – Incidence difference per 1000 person-years.

| **COVID-19 versus Influenza Female** | | | | | | |
| --- | --- | --- | --- | --- | --- | --- |
|  | 3 to 6 months after index date | | | 12 to 15 months after index date | | |
| **Symptom or Condition** | IRR | 95% CI | IR difference | IRR | 95% CI | IR difference |
| **WHO post-COVID** |  |  |  |  |  |  |
| WHO post-COVID Definition | 1.74 | 1.70-1.79 | 62.67 | 1.31 | 1.25-1.37 | 29.12 |
| Malaise/exhaustion | 1.45 | 1.40-1.50 | 20.50 | 1.36 | 1.27-1.46 | 16.28 |
| Chronic fatigue syndrome | 7.04 | 6.12-8.11 | 10.33 | 1.15 | 0.99-1.33 | 1.44 |
| Dyspnea | 2.74 | 2.62-2.87 | 39.39 | 1.71 | 1.57-1.86 | 18.35 |
| Respiratory insufficiency | 1.41 | 1.30-1.52 | 3.60 | 1.31 | 1.11-1.55 | 2.24 |
| Chest pain | 2.83 | 2.01-3.99 | 0.63 | 3.63 | 1.72-7.65 | 0.66 |
| Cognitive Impairment | 1.23 | 1.14-1.32 | 2.27 | 1.26 | 1.08-1.47 | 2.29 |
| Memory disorder | 2.26 | 2.03-2.53 | 4.60 | 0.90 | 0.75-1.07 | -0.77 |
| **Tissue damage** |  |  |  |  |  |  |
| Pulmonary embolism | 2.91 | 2.50-3.40 | 3.28 | 1.69 | 1.33-2.16 | 2.06 |
| Lung damage | 2.80 | 2.27-3.45 | 2.56 | 2.76 | 1.78-4.28 | 2.15 |
| Pericarditis | 3.27 | 2.17-4.95 | 0.52 | 3.49 | 1.71-7.09 | 0.69 |
| Myocarditis | 3.42 | 2.01-5.81 | 0.46 | 5.18 | 1.76-15.21 | 0.52 |
| **Control endpoints** |  |  |  |  |  |  |
| Melanoma | 0.49 | 0.42-0.58 | -1.84 | 0.48 | 0.36-0.64 | -2.25 |
| Tinea pedis | 1.59 | 1.37-1.85 | 1.32 | 1.01 | 0.80-1.28 | 0.04 |
| **COVID-19 versus Influenza Male** | | | | | | |
|  | 3 to 6 months after index date | | | 12 to 15 months after index date | | |
| **Symptom or Condition** | IRR | 95% CI | IR difference | IRR | 95% CI | IR difference |
| **WHO post-COVID** |  |  |  |  |  |  |
| WHO post-COVID Definition | 2.16 | 2.09-2.23 | 64.58 | 1.50 | 1.40-1.59 | 34.23 |
| Malaise/exhaustion | 2.30 | 2.17-2.43 | 25.30 | 1.81 | 1.62-2.02 | 19.28 |
| Chronic fatigue syndrome | 13.92 | 10.34-18.72 | 6.59 | 1.34 | 1.05-1.72 | 1.65 |
| Dyspnea | 2.51 | 2.38-2.66 | 30.56 | 1.39 | 1.25-1.55 | 10.66 |
| Respiratory insufficiency | 1.96 | 1.80-2.14 | 8.39 | 1.85 | 1.53-2.25 | 6.13 |
| Chest pain | 11.18 | 5.42-23.09 | 0.88 | 1.75 | 0.79-3.85 | 0.32 |
| Cognitive Impairment | 1.12 | 1.02-1.23 | 1.09 | 0.96 | 0.80-1.15 | -0.45 |
| Memory disorder | 2.28 | 1.97-2.64 | 3.74 | 2.45 | 1.82-3.30 | 3.84 |
| **Tissue damage** |  |  |  |  |  |  |
| Pulmonary embolism | 2.29 | 1.98-2.65 | 3.68 | 2.51 | 1.82-3.45 | 3.47 |
| Lung damage | 1.75 | 1.49-2.06 | 1.90 | 2.28 | 1.60-3.25 | 2.45 |
| Pericarditis | 1.11 | 0.75-1.63 | 0.06 | 1.09 | 0.38-3.11 | 0.03 |
| Myocarditis | 1.81 | 1.15-2.84 | 0.26 | 2.50 | 1.03-6.07 | 0.44 |
| **Negative control endpoints** |  |  |  |  |  |  |
| Melanoma | 0.97 | 0.77-1.22 | -0.05 | 1.62 | 0.97-2.68 | 0.66 |
| Tinea pedis | 0.89 | 0.79-1.00 | -0.70 | 0.80 | 0.64-0.99 | -1.64 |

Table5 Estimates of Poisson regression of COVID-19 compared to the matched Non-COVID contemporary control and influenza cohort 3 to 6 and 12 to 15 months after infection for outpatient patients. IRR – Incidence rate ratios, IR – Incidence difference per 1000 person-years.

| **COVID-19 versus Non-COVID Outpatient** | | | | | | |
| --- | --- | --- | --- | --- | --- | --- |
|  | 3 to 6 months after index date | | | 12 to 15 months after index date | | |
| **Symptom or Condition** | IRR | 95% CI | IR difference | IRR | 95% CI | IR difference |
| **WHO post-COVID** |  |  |  |  |  |  |
| WHO post-COVID Definition | 2.04 | 2.00-2.09 | 62.38 | 1.57 | 1.51-1.64 | 40.73 |
| Malaise/exhaustion | 1.87 | 1.81-1.93 | 25.73 | 1.58 | 1.49-1.68 | 20.23 |
| Chronic fatigue syndrome | 5.22 | 4.65-5.86 | 7.41 | 3.06 | 2.53-3.69 | 5.66 |
| Dyspnea | 2.94 | 2.83-3.06 | 35.09 | 1.96 | 1.82-2.11 | 19.76 |
| Respiratory insufficiency | 1.47 | 1.36-1.59 | 2.73 | 1.27 | 1.11-1.47 | 1.85 |
| Chest pain | 2.33 | 1.79-3.04 | 0.54 | 1.60 | 0.97-2.64 | 0.29 |
| Cognitive Impairment | 1.15 | 1.07-1.24 | 1.11 | 1.10 | 0.97-1.25 | 0.88 |
| Memory disorder | 1.62 | 1.47-1.78 | 2.26 | 1.26 | 1.06-1.49 | 1.18 |
| **Tissue damage** |  |  |  |  |  |  |
| Pulmonary embolism | 2.08 | 1.81-2.39 | 1.67 | 1.85 | 1.47-2.33 | 1.80 |
| Lung damage | 2.33 | 1.95-2.79 | 1.18 | 1.59 | 1.18-2.13 | 0.81 |
| Pericarditis | 3.34 | 2.27-4.92 | 0.40 | 2.26 | 1.24-4.11 | 0.37 |
| Myocarditis | 3.41 | 2.17-5.34 | 0.31 | 3.49 | 1.68-7.27 | 0.44 |
| **Control endpoints** |  |  |  |  |  |  |
| Melanoma | 1.21 | 1.03-1.42 | 0.28 | 0.92 | 0.70-1.20 | -0.17 |
| Tinea pedis | 1.27 | 1.15-1.41 | 0.93 | 1.24 | 1.04-1.49 | 0.98 |
| **COVID-19 versus Influenza Outpatient** | | | | | | |
|  | 3 to 6 months after index date | | | 12 to 15 months after index date | | |
| **Symptom or Condition** | IRR | 95% CI | IR difference | IRR | 95% CI | IR difference |
| **WHO post-COVID** |  |  |  |  |  |  |
| WHO post-COVID Definition | 1.93 | 1.76-2.11 | 56.91 | 1.39 | 1.27-1.53 | 29.97 |
| Malaise/exhaustion | 1.68 | 1.48-1.92 | 21.85 | 1.50 | 1.30-1.73 | 17.41 |
| Chronic fatigue syndrome | 8.71 | 4.34-17.49 | 8.08 | 1.06 | 0.79-1.42 | 0.44 |
| Dyspnea | 2.63 | 2.23-3.10 | 32.19 | 1.61 | 1.36-1.90 | 14.63 |
| Respiratory insufficiency | 1.46 | 1.06-2.00 | 2.43 | 1.75 | 1.20-2.55 | 3.50 |
| Chest pain | 4.34 | 0.94-20.07 | 0.72 | 3.29 | 0.63-17.16 | 0.53 |
| Cognitive Impairment | 1.15 | 0.87-1.53 | 0.99 | 1.24 | 0.91-1.69 | 1.72 |
| Memory disorder | 2.91 | 1.74-4.87 | 3.71 | 1.24 | 0.84-1.82 | 1.08 |
| **Tissue damage** |  |  |  |  |  |  |
| Pulmonary embolism | 2.31 | 1.24-4.29 | 1.77 | 2.08 | 1.17-3.72 | 2.07 |
| Lung damage | 1.77 | 0.91-3.47 | 0.89 | 2.52 | 1.06-5.97 | 1.28 |
| Pericarditis | 3.62 | 0.62-21.11 | 0.43 | 2.56 | 0.55-12.01 | 0.41 |
| Myocarditis | 2.90 | 0.47-17.92 | 0.29 | 4.25 | 0.52-34.64 | 0.46 |
| **Negative control endpoints** |  |  |  |  |  |  |
| Melanoma | 0.55 | 0.36-0.85 | -1.28 | 0.58 | 0.36-0.93 | -1.34 |
| Tinea pedis | 1.16 | 0.79-1.69 | 0.57 | 0.90 | 0.63-1.29 | -0.52 |

Table6 Estimates of Poisson regression of COVID-19 compared to the matched Non-COVID contemporary control and influenza cohort 3 to 6 and 12 to 15 months after infection for hospitalized non-ICU patients. IRR – Incidence rate ratios, IR – Incidence difference per 1000 person-years.

| **COVID-19 versus Non-COVID Hospital** | | | | | | |
| --- | --- | --- | --- | --- | --- | --- |
|  | 3 to 6 months after index date | | | 12 to 15 months after index date | | |
| **Symptom or Condition** | IRR | 95% CI | IR difference | IRR | 95% CI | IR difference |
| **WHO post-COVID** |  |  |  |  |  |  |
| WHO post-COVID Definition | 2.62 | 2.48-2.78 | 192.39 | 2.01 | 1.75-2.31 | 120.58 |
| Malaise/exhaustion | 2.74 | 2.45-3.06 | 58.22 | 2.21 | 1.70-2.87 | 43.80 |
| Chronic fatigue syndrome | 8.19 | 5.65-11.88 | 16.48 | 11.56 | 4.33-30.84 | 18.69 |
| Dyspnea | 3.25 | 2.92-3.61 | 81.05 | 2.46 | 1.89-3.22 | 49.32 |
| Respiratory insufficiency | 3.23 | 2.84-3.67 | 53.53 | 1.92 | 1.39-2.65 | 22.54 |
| Chest pain | 2.21 | 0.89-5.45 | 0.60 | 3.51 | 0.73-16.87 | 2.04 |
| Cognitive Impairment | 2.19 | 1.93-2.48 | 34.12 | 1.59 | 1.15-2.18 | 15.94 |
| Memory disorder | 1.94 | 1.63-2.30 | 14.01 | 1.63 | 1.06-2.50 | 8.93 |
| **Tissue damage** |  |  |  |  |  |  |
| Pulmonary embolism | 6.64 | 5.05-8.73 | 24.82 | 3.87 | 2.20-6.83 | 17.81 |
| Lung damage | 4.64 | 3.33-6.46 | 11.40 | 6.74 | 2.75-16.50 | 12.93 |
| Pericarditis | 8.44 | 2.71-26.29 | 1.82 | 4.81 | 0.45-50.92 | 1.29 |
| Myocarditis | 14.07 | 1.85-107.02 | 0.96 | 9.01 | 0.25-322.83 | 1.09 |
| **Control endpoints** |  |  |  |  |  |  |
| Melanoma | 1.47 | 0.94-2.29 | 1.14 | 0.83 | 0.32-2.12 | -0.68 |
| Tinea pedis | 1.06 | 0.79-1.43 | 0.38 | 1.05 | 0.47-2.35 | 0.21 |
| **COVID-19 versus Influenza Hospital** | | | | | | |
|  | 3 to 6 months after index date | | | 12 to 15 months after index date | | |
| **Symptom or Condition** | IRR | 95% CI | IR difference | IRR | 95% CI | IR difference |
| **WHO post-COVID** |  |  |  |  |  |  |
| WHO post-COVID Definition | 1.56 | 1.44-1.69 | 108.19 | 1.13 | 0.99-1.30 | 27.15 |
| Malaise/exhaustion | 1.41 | 1.22-1.64 | 26.15 | 1.42 | 1.07-1.89 | 22.31 |
| Chronic fatigue syndrome | 4.74 | 2.76-8.14 | 14.46 | 2.76 | 1.43-5.33 | 12.92 |
| Dyspnea | 2.60 | 2.18-3.09 | 71.35 | 1.15 | 0.89-1.49 | 10.51 |
| Respiratory insufficiency | 1.59 | 1.34-1.89 | 27.20 | 1.17 | 0.84-1.63 | 6.84 |
| Chest pain | 2.14 | 0.51-8.90 | 0.67 | 1.92 | 0.38-9.78 | 1.18 |
| Cognitive Impairment | 1.18 | 1.00-1.40 | 9.04 | 0.82 | 0.60-1.12 | -9.41 |
| Memory disorder | 1.45 | 1.13-1.87 | 9.26 | 1.08 | 0.70-1.67 | 1.78 |
| **Tissue damage** |  |  |  |  |  |  |
| Pulmonary embolism | 2.75 | 1.96-3.85 | 18.34 | 1.85 | 1.08-3.16 | 10.48 |
| Lung damage | 2.81 | 1.73-4.55 | 9.23 | 2.36 | 1.16-4.82 | 8.91 |
| Pericarditis | 0.68 | 0.33-1.38 | -0.95 | 1.92 | 0.26-14.11 | 0.79 |
| Myocarditis | 2.35 | 0.46-12.09 | 0.59 | 1.44 | 0.18-11.45 | 0.38 |
| **Negative control endpoints** |  |  |  |  |  |  |
| Melanoma | 1.35 | 0.67-2.73 | 0.92 | 4.32 | 0.67-27.64 | 2.88 |
| Tinea pedis | 0.81 | 0.53-1.25 | -1.49 | 0.59 | 0.28-1.27 | -3.77 |

Table7 Estimates of Poisson regression of COVID-19 compared to the matched Non-COVID contemporary control and influenza cohort 3 to 6 and 12 to 15 months after infection for hospitalized ICU patients. IRR – Incidence rate ratios, IR – Incidence difference per 1000 person-years.

| **COVID-19 versus Non-COVID ICU** | | | | | | |
| --- | --- | --- | --- | --- | --- | --- |
|  | 3 to 6 months after index date | | | 12 to 15 months after index date | | |
| **Symptom or Condition** | IRR | 95% CI | IR difference | IRR | 95% CI | IR difference |
| **WHO post-COVID** |  |  |  |  |  |  |
| WHO post-COVID Definition | 4.40 | 3.94-4.91 | 400.00 | 3.44 | 2.71-4.36 | 304.26 |
| Malaise/exhaustion | 3.96 | 3.16-4.96 | 91.46 | 3.56 | 2.13-5.93 | 73.25 |
| Chronic fatigue syndrome | 12.83 | 5.94-27.69 | 25.00 | 14.99 | 3.98-56.41 | 46.36 |
| Dyspnea | 4.79 | 3.91-5.86 | 144.64 | 4.31 | 2.80-6.63 | 132.08 |
| Respiratory insufficiency | 7.62 | 6.14-9.45 | 204.67 | 5.73 | 3.29-9.97 | 106.29 |
| Chest pain | 2.33 | 0.47-11.47 | 0.87 | 2.00 | 0.07-59.58 | 0.71 |
| Cognitive Impairment | 3.17 | 2.47-4.08 | 56.55 | 2.02 | 1.12-3.66 | 25.18 |
| Memory disorder | 2.97 | 2.10-4.20 | 26.16 | 1.36 | 0.72-2.59 | 8.49 |
| **Tissue damage** |  |  |  |  |  |  |
| Pulmonary embolism | 10.73 | 6.82-16.88 | 61.13 | 10.08 | 3.45-29.49 | 47.84 |
| Lung damage | 12.29 | 6.65-22.73 | 37.68 | 5.76 | 2.03-16.29 | 28.32 |
| Pericarditis | - | - | - | - | - | - |
| Myocarditis | - | - | - | - | - | - |
| **Control endpoints** |  |  |  |  |  |  |
| Melanoma | 1.42 | 0.49-4.18 | 0.73 | 1.15 | 0.32-4.17 | 0.95 |
| Tinea pedis | 0.95 | 0.54-1.67 | -0.39 | 1.50 | 0.42-5.31 | 2.86 |
| **COVID-19 versus Influenza ICU** | | | | | | |
|  | 3 to 6 months after index date | | | 12 to 15 months after index date | | |
| **Symptom or Condition** | IRR | 95% CI | IR difference | IRR | 95% CI | IR difference |
| **WHO post-COVID** |  |  |  |  |  |  |
| WHO post-COVID Definition | 1.80 | 1.56-2.08 | 230.75 | 1.15 | 0.93-1.42 | 55.26 |
| Malaise/exhaustion | 1.56 | 1.15-2.11 | 40.96 | 0.98 | 0.63-1.53 | -1.50 |
| Chronic fatigue syndrome | 40.38 | 2.49-654.49 | 27.09 | - | - | - |
| Dyspnea | 2.94 | 2.13-4.06 | 125.16 | 1.74 | 1.12-2.68 | 69.43 |
| Respiratory insufficiency | 2.08 | 1.64-2.65 | 129.41 | 0.90 | 0.62-1.32 | -14.06 |
| Chest pain | - | - | - | - | - | - |
| Cognitive Impairment | 1.12 | 0.83-1.51 | 9.21 | 0.62 | 0.37-1.04 | -32.83 |
| Memory disorder | 1.38 | 0.86-2.21 | 11.20 | 0.64 | 0.32-1.29 | -15.90 |
| **Tissue damage** |  |  |  |  |  |  |
| Pulmonary embolism | 2.82 | 1.73-4.61 | 44.84 | 1.22 | 0.65-2.29 | 9.43 |
| Lung damage | 2.43 | 1.33-4.43 | 23.38 | 1.77 | 0.69-4.55 | 12.93 |
| Pericarditis | - | - | - | - | - | - |
| Myocarditis | - | - | - | - | - | - |
| **Negative control endpoints** |  |  |  |  |  |  |
| Melanoma | - | - | - | - | - | - |
| Tinea pedis | 2.39 | 0.56-10.20 | 3.94 | 1.23 | 0.26-5.87 | 1.54 |

Table8 Estimates of Poisson regression of COVID-19 compared to the matched Non-COVID contemporary control 3 to 6 and 12 to 15 months after infection by age groups. IRR – Incidence rate ratios, IR – Incidence difference per 1000 person-years.

| **COVID-19 versus Non-COVID Age group 18-29** | | | | | | |
| --- | --- | --- | --- | --- | --- | --- |
|  | 3 to 6 months after index date | | | 12 to 15 months after index date | | |
| Symptom or Condition | IRR | 95% CI | IR difference | IRR | 95% CI | IR difference |
| **WHO post-COVID** |  |  |  |  |  |  |
| WHO post-COVID Definition | 2.07 | 1.96-2.19 | 48.56 | 1.61 | 1.45-1.79 | 33.51 |
| Malaise/exhaustion | 1.72 | 1.60-1.85 | 22.24 | 1.51 | 1.32-1.72 | 19.28 |
| Chronic fatigue syndrome | 3.93 | 2.89-5.34 | 3.63 | 2.24 | 1.31-3.84 | 2.33 |
| Dyspnea | 3.19 | 2.89-3.53 | 26.94 | 2.15 | 1.75-2.65 | 15.11 |
| Respiratory insufficiency | 2.86 | 2.00-4.11 | 1.80 | 1.79 | 0.91-3.55 | 1.00 |
| Chest pain | 1.91 | 1.10-3.32 | 0.43 | 5.14 | 1.27-20.91 | 0.95 |
| Cognitive Impairment | 1.33 | 0.96-1.84 | 0.51 | 1.30 | 0.75-2.26 | 0.65 |
| Memory disorder | 2.88 | 1.93-4.29 | 1.47 | 1.67 | 0.81-3.41 | 0.78 |
| **Tissue damage** |  |  |  |  |  |  |
| Pulmonary embolism | 3.74 | 1.87-7.47 | 0.67 | 1.50 | 0.47-4.84 | 0.23 |
| Lung damage | 3.43 | 1.36-8.62 | 0.34 | 1.15 | 0.32-4.18 | 0.07 |
| Pericarditis | 2.14 | 0.87-5.26 | 0.19 | 7.50 | 0.58-96.60 | 0.42 |
| Myocarditis | 1.75 | 0.73-4.17 | 0.15 | 6.00 | 1.16-30.93 | 0.81 |
| **Control endpoints** |  |  |  |  |  |  |
| Melanoma | 1.17 | 0.62-2.19 | 0.07 | 0.71 | 0.23-2.25 | -0.20 |
| Tinea pedis | 1.13 | 0.87-1.47 | 0.32 | 0.87 | 0.52-1.47 | -0.38 |
| **COVID-19 versus Non-COVID Age group 30-39** | | | | | | |
|  | 3 to 6 months after index date | | | 12 to 15 months after index date | | |
| **Symptom or Condition** | IRR | 95% CI | IR difference | IRR | 95% CI | IR difference |
| **WHO post-COVID** |  |  |  |  |  |  |
| WHO post-COVID Definition | 2.28 | 2.14-2.42 | 59.80 | 1.81 | 1.61-2.03 | 42.80 |
| Malaise/exhaustion | 1.88 | 1.73-2.04 | 27.86 | 1.67 | 1.44-1.93 | 24.02 |
| Chronic fatigue syndrome | 5.91 | 4.35-8.05 | 7.54 | 3.17 | 1.86-5.38 | 4.56 |
| Dyspnea | 3.66 | 3.26-4.12 | 31.31 | 2.56 | 2.05-3.21 | 19.73 |
| Respiratory insufficiency | 2.39 | 1.64-3.48 | 1.73 | 2.59 | 1.23-5.43 | 1.79 |
| Chest pain | 3.17 | 1.65-6.06 | 0.84 | 1.35 | 0.50-3.67 | 0.27 |
| Cognitive Impairment | 1.55 | 1.14-2.13 | 1.16 | 1.48 | 0.90-2.44 | 1.44 |
| Memory disorder | 2.89 | 1.86-4.48 | 1.63 | 2.68 | 1.08-6.68 | 1.24 |
| **Tissue damage** |  |  |  |  |  |  |
| Pulmonary embolism | 3.20 | 1.94-5.26 | 1.44 | 3.68 | 1.39-9.72 | 1.61 |
| Lung damage | 3.17 | 1.26-7.93 | 0.42 | 1.50 | 0.38-6.00 | 0.19 |
| Pericarditis | 11.00 | 2.59-46.77 | 0.65 | 5.00 | 0.58-42.78 | 0.47 |
| Myocarditis | 6.00 | 1.83-19.71 | 0.51 | 8.40 | 0.87-81.38 | 0.72 |
| **Negative control endpoints** |  |  |  |  |  |  |
| Melanoma |  | 1.41 0.75 2.65 | 0.22  0.56 |  | 1.67 0.66 4.22 |  |
| Tinea pedis |  | 1.49 1.11 2.00 | 1.16  2.26 |  | 1.67 1.05 2.66 |  |
| **COVID-19 versus Non-COVID Age group 40-49** | | | | | | |
|  | 3 to 6 months after index date | | | 12 to 15 months after index date | | |
| **Symptom or Condition** | IRR | 95% CI | IR difference | IRR | 95% CI | IR difference |
| **WHO post-COVID** |  |  |  |  |  |  |
| WHO post-COVID Definition | 2.61 | 2.47-2.75 | 79.26 | 1.72 | 1.55-1.90 | 41.85 |
| Malaise/exhaustion | 1.97 | 1.83-2.12 | 30.37 | 1.51 | 1.31-1.73 | 18.25 |
| Chronic fatigue syndrome | 5.59 | 4.35-7.17 | 9.05 | 3.54 | 2.32-5.38 | 7.20 |
| Dyspnea | 4.41 | 4.00-4.86 | 47.37 | 2.36 | 1.96-2.85 | 21.94 |
| Respiratory insufficiency | 2.10 | 1.61-2.76 | 2.33 | 1.42 | 0.88-2.27 | 1.25 |
| Chest pain | 2.91 | 1.67-5.06 | 0.87 | 1.55 | 0.51-4.68 | 0.29 |
| Cognitive Impairment | 1.58 | 1.24-2.02 | 1.64 | 1.06 | 0.66-1.70 | 0.19 |
| Memory disorder | 3.94 | 2.76-5.63 | 3.02 | 1.47 | 0.79-2.72 | 0.81 |
| **Tissue damage** |  |  |  |  |  |  |
| Pulmonary embolism | 2.73 | 1.89-3.95 | 1.81 | 2.67 | 1.43-4.99 | 2.28 |
| Lung damage | 3.92 | 2.28-6.75 | 1.29 | 1.29 | 0.55-3.02 | 0.27 |
| Pericarditis | 4.55 | 1.81-11.44 | 0.53 | 1.78 | 0.56-5.64 | 0.35 |
| Myocarditis | 6.21 | 2.34-16.52 | 0.66 | 2.00 | 0.44-9.13 | 0.25 |
| **Negative control endpoints** |  |  |  |  |  |  |
| Melanoma | 1.46 | 1.00-2.13 | 0.58 | 0.70 | 0.34-1.41 | -0.57 |
| Tinea pedis | 1.39 | 1.10-1.75 | 1.29 | 1.46 | 0.98-2.19 | 1.87 |
| **COVID-19 versus Non-COVID Age group 50-59** | | | | | | |
|  | 3 to 6 months after index date | | | 12 to 15 months after index date | | |
| **Symptom or Condition** | IRR | 95% CI | IR difference | IRR | 95% CI | IR difference |
| **WHO post-COVID** |  |  |  |  |  |  |
| WHO post-COVID Definition | 2.61 | 2.50-2.74 | 87.80 | 1.87 | 1.72-2.04 | 56.68 |
| Malaise/exhaustion | 2.12 | 1.98-2.27 | 31.70 | 1.72 | 1.52-1.95 | 23.77 |
| Chronic fatigue syndrome | 6.09 | 5.00-7.41 | 12.81 | 3.98 | 2.90-5.48 | 11.01 |
| Dyspnea | 3.81 | 3.53-4.12 | 50.99 | 2.20 | 1.90-2.54 | 25.63 |
| Respiratory insufficiency | 2.23 | 1.89-2.64 | 5.31 | 1.60 | 1.17-2.19 | 2.94 |
| Chest pain | 2.68 | 1.46-4.94 | 0.52 | 1.14 | 0.41-3.15 | 0.08 |
| Cognitive Impairment | 1.81 | 1.51-2.17 | 3.20 | 1.49 | 1.09-2.04 | 2.46 |
| Memory disorder | 3.36 | 2.64-4.27 | 4.38 | 2.12 | 1.39-3.23 | 2.75 |
| **Tissue damage** |  |  |  |  |  |  |
| Pulmonary embolism | 3.26 | 2.52-4.21 | 3.72 | 2.48 | 1.60-3.85 | 3.22 |
| Lung damage | 3.12 | 2.27-4.28 | 2.33 | 2.09 | 1.20-3.66 | 1.54 |
| Pericarditis | 5.50 | 2.59-11.68 | 0.78 | 2.64 | 0.85-8.15 | 0.53 |
| Myocarditis | 5.79 | 2.21-15.21 | 0.50 | 7.71 | 1.12-53.05 | 0.61 |
| **Negative control endpoints** |  |  |  |  |  |  |
| Melanoma | 1.26 | 0.93-1.70 | 0.42 | 0.88 | 0.55-1.39 | -0.38 |
| Tinea pedis | 1.24 | 1.01-1.52 | 0.90 | 1.11 | 0.77-1.60 | 0.47 |
| **COVID-19 versus Non-COVID Age group 60-69** | | | | | | |
|  | 3 to 6 months after index date | | | 12 to 15 months after index date | | |
| **Symptom or Condition** | IRR | 95% CI | IR difference | IRR | 95% CI | IR difference |
| **WHO post-COVID** |  |  |  |  |  |  |
| WHO post-COVID Definition | 2.60 | 2.45-2.75 | 99.55 | 1.88 | 1.69-2.09 | 66.12 |
| Malaise/exhaustion | 2.53 | 2.28-2.80 | 33.04 | 2.02 | 1.68-2.44 | 25.87 |
| Chronic fatigue syndrome | 8.00 | 5.79-11.03 | 11.65 | 4.36 | 2.70-7.03 | 10.04 |
| Dyspnea | 2.95 | 2.69-3.23 | 50.05 | 2.22 | 1.86-2.64 | 33.85 |
| Respiratory insufficiency | 2.79 | 2.40-3.25 | 16.75 | 1.76 | 1.33-2.31 | 8.87 |
| Chest pain | 2.61 | 1.20-5.66 | 0.56 | 3.00 | 0.42-21.30 | 0.38 |
| Cognitive Impairment | 1.80 | 1.51-2.15 | 6.07 | 1.21 | 0.86-1.72 | 1.80 |
| Memory disorder | 2.20 | 1.73-2.81 | 4.51 | 1.83 | 1.19-2.80 | 3.87 |
| **Tissue damage** |  |  |  |  |  |  |
| Pulmonary embolism | 4.35 | 3.34-5.66 | 9.07 | 2.84 | 1.79-4.49 | 6.58 |
| Lung damage | 3.80 | 2.72-5.29 | 4.92 | 3.37 | 1.84-6.16 | 4.68 |
| Pericarditis | 2.62 | 1.06-6.46 | 0.42 | 2.80 | 0.66-11.87 | 0.65 |
| Myocarditis | 3.00 | 0.81-11.09 | 0.24 | 1.00 | 0.06-15.99 | 0.00 |
| **Negative control endpoints** |  |  |  |  |  |  |
| Melanoma | 1.18 | 0.81-1.73 | 0.36 | 1.12 | 0.62-2.00 | 0.36 |
| Tinea pedis | 1.27 | 1.00-1.62 | 1.26 | 1.19 | 0.77-1.85 | 1.05 |
| **COVID-19 versus Non-COVID Age group 70-79** | | | | | | |
|  | 3 to 6 months after index date | | | 12 to 15 months after index date | | |
| **Symptom or Condition** | IRR | 95% CI | IR difference | IRR | 95% CI | IR difference |
| **WHO post-COVID** |  |  |  |  |  |  |
| WHO post-COVID Definition | 1.85 | 1.74-1.97 | 97.63 | 1.44 | 1.28-1.62 | 61.25 |
| Malaise/exhaustion | 2.20 | 1.93-2.51 | 29.26 | 1.66 | 1.30-2.13 | 20.87 |
| Chronic fatigue syndrome | 3.94 | 2.56-6.08 | 5.47 | 3.63 | 1.66-7.93 | 6.17 |
| Dyspnea | 1.98 | 1.78-2.21 | 37.92 | 1.64 | 1.32-2.03 | 28.07 |
| Respiratory insufficiency | 2.33 | 2.05-2.66 | 33.09 | 1.65 | 1.25-2.18 | 16.23 |
| Chest pain | 1.15 | 0.44-3.00 | 0.09 | 1.26 | 0.29-5.52 | 0.24 |
| Cognitive Impairment | 1.37 | 1.21-1.56 | 11.37 | 1.12 | 0.87-1.43 | 4.46 |
| Memory disorder | 1.44 | 1.21-1.71 | 6.96 | 1.09 | 0.79-1.51 | 2.03 |
| **Tissue damage** |  |  |  |  |  |  |
| Pulmonary embolism | 3.40 | 2.58-4.49 | 11.37 | 1.94 | 1.18-3.21 | 6.51 |
| Lung damage | 3.28 | 2.40-4.49 | 8.47 | 2.56 | 1.47-4.48 | 7.94 |
| Pericarditis | 2.89 | 0.95-8.80 | 0.57 | 2.00 | 0.28-14.21 | 0.44 |
| Myocarditis | 4.21 | 0.78-22.80 | 0.39 | 2.00 | 0.37-10.93 | 0.59 |
| **Negative control endpoints** |  |  |  |  |  |  |
| Melanoma | 0.96 | 0.64-1.43 | -0.15 | 0.86 | 0.41-1.80 | -0.64 |
| Tinea pedis | 1.18 | 0.89-1.57 | 1.14 | 1.02 | 0.56-1.84 | 0.10 |
| **COVID-19 versus Non-COVID Age group 80-89** | | | | | | |
|  | 3 to 6 months after index date | | | 12 to 15 months after index date | | |
| **Symptom or Condition** | IRR | 95% CI | IR difference | IRR | 95% CI | IR difference |
| **WHO post-COVID** |  |  |  |  |  |  |
| WHO post-COVID Definition | 1.58 | 1.50-1.67 | 95.33 | 1.23 | 1.10-1.38 | 44.21 |
| Malaise/exhaustion | 1.84 | 1.64-2.06 | 32.39 | 1.49 | 1.17-1.91 | 21.20 |
| Chronic fatigue syndrome | 3.94 | 2.43-6.39 | 4.74 | 1.71 | 0.68-4.36 | 1.82 |
| Dyspnea | 1. 50 | 1.35-1.68 | 23.43 | 1.31 | 1.04-1.66 | 15.98 |
| Respiratory insufficiency | 2.06 | 1.83-2.30 | 38.75 | 1.41 | 1.09-1.81 | 16.21 |
| Chest pain | 1.26 | 0.47-3.37 | 0.15 | 2.25 | 0.29-17.31 | 0.61 |
| Cognitive Impairment | 1.40 | 1.26-1.55 | 22.33 | 1.12 | 0.90-1.39 | 8.17 |
| Memory disorder | 1.28 | 1.10-1.47 | 7.68 | 1.02 | 0.76-1.37 | 0.60 |
| **Tissue damage** |  |  |  |  |  |  |
| Pulmonary embolism | 2.80 | 2.17-3.61 | 11.58 | 1.79 | 1.10-2.91 | 7.38 |
| Lung damage | 2.86 | 2.02-4.04 | 6.31 | 1.56 | 0.79-3.08 | 2.76 |
| Pericarditis | - | - | - | - | - | - |
| Myocarditis | - | - | - | - | - | - |
| **Negative control endpoints** |  |  |  |  |  |  |
| Melanoma | 1.15 | 0.76-1.75 | 0.49 | 0.76 | 0.33-1.73 | -1.16 |
| Tinea pedis | 0.96 | 0.70-1.32 | -0.24 | 1.27 | 0.59-2.72 | 1.17 |
| **COVID-19 versus Non-COVID Age group 90+** | | | | | | |
|  | 3 to 6 months after index date | | | 12 to 15 months after index date | | |
| **Symptom or Condition** | IRR | 95% CI | IR difference | IRR | 95% CI | IR difference |
| **WHO post-COVID** |  |  |  |  |  |  |
| WHO post-COVID Definition | 1.19 | 1.07-1.31 | 35.40 | 0.99 | 0.80-1.23 | -1.72 |
| Malaise/exhaustion | 1.30 | 1.07-1.58 | 16.32 | 1.40 | 0.93-2.11 | 22.45 |
| Chronic fatigue syndrome | 3.17 | 0.90-11.23 | 1.84 | 2.74 | 0.50-14.89 | 4.04 |
| Dyspnea | 1.33 | 1.07-1.64 | 14.55 | 0.92 | 0.59-1.44 | -4.51 |
| Respiratory insufficiency | 1.40 | 1.15-1.71 | 19.82 | 0.74 | 0.47-1.16 | -15.91 |
| Chest pain | - | - | - | - | - | - |
| Cognitive Impairment | 1.06 | 0.87-1.28 | 3.77 | 1.00 | 0.65-1.55 | 0.18 |
| Memory disorder | 0.87 | 0.67-1.13 | -4.77 | 0.71 | 0.41-1.24 | -12.07 |
| **Tissue damage** |  |  |  |  |  |  |
| Pulmonary embolism | 1.82 | 1.04-3.17 | 4.28 | 1.66 | 0.54-5.15 | 4.14 |
| Lung damage | 1.44 | 0.57-3.63 | 0.91 | 2.01 | 0.37-10.97 | 2.57 |
| Pericarditis | - | - | - | - | - | - |
| Myocarditis | - | - | - | - | - | - |
| **Negative control endpoints** |  |  |  |  |  |  |
| Melanoma | 2.30 | 0.95-5.58 | 2.43 | 1.29 | 0.23-7.15 | 0.87 |
| Tinea pedis | 1.09 | 0.57-2.09 | 0.43 | 2.15 | 0.46-10.19 | 3.45 |
